# Data on biochemical fluxes generated from biofabricated enzyme complexes assembled through engineered tags and microbial transglutaminase

**DOI:** 10.1016/j.dib.2016.07.005

**Published:** 2016-07-09

**Authors:** Narendranath Bhokisham, Haig Pakhchanian, David Quan, Tanya Tschirhart, Chen-Yu Tsao, Gregory F. Payne, William E. Bentley

**Affiliations:** a Biological Sciences Graduate Program - College of Computer, Mathematical and Natural Sciences, University of Maryland, 4066 Campus Drive, College Park, MD 20742, United States; b Institute of Bioscience and Biotechnology Research, University of Maryland, College Park, 5115 Plant Science and Landscape Architecture Building, College Park, MD 20742, United States; c Fischell Department of Bioengineering, University of Maryland, Room 3122, Jeong H. Kim Engineering Building (Bldg. #225), College Park, MD 20742, United States

**Keywords:** Biofabrication, Metabolic flux, Engineered tags, Transglutaminase, Quorum sensing

## Abstract

Data presented is related to an article titled “*Modular construction of multi-subunit protein complexes using engineered tags and microbial transglutaminase*” (Bhokisham et al., 2016) [1]. In this article, we have presented western blot and flux data associated with assembly of Pfs–LuxS enzyme complexes on beads using uni-tagged and bi-tagged LuxS enzymes. We have also presented biochemical flux following changes in enzyme stoichiometries. We covalently coupled a Pfs-LuxS complex with Protein G, an antibody binding non-enzyme component and directed these complexes to the surfaces of bacterial cells via anti-*Escherichia coli* antibodies. Fluorescence microscopy images represented the altered behavior of bacterial cells in response to the autoinducer-2 that is synthesized by the Protein G-enzyme complexes.

## Specifications Table

**Table:** 

Subject area	Chemistry and Biology
More specific subject area	Biotechnology; Metabolic Engineering
Type of data	Graphs and Images
How data was acquired	Calorimetric Measurements, Western Blotting and Fluorescence Microscopy
Data format	Analyzed
Experimental factors	We stored engineered protein components at −20 °C until further use. We performed each round of crosslinking on beads at RT for 60 min. We incubated enzyme components with substrates at 37 °C for measurement of flux. For translocation experiments, we grew bacterial cells at 30 °C overnight. We assembled protein components onto bacterial cells by incubating at RT for 60 min and after assembly, we incubated both cells and protein components with substrate, SAH, at 30 °C for 4 h without shaking.
Experimental features	We presented western blotting data to indicate protein crosslinking. We used flux data from various QS metabolons to depict the enzyme activities of assembled complexes. We also showed the metabolic response of bacteria in response to metabolons.
Data source location	University of Maryland, College Park, MD, USA.
Data accessibility	Data is within this article.

## Value of the data

• Data demonstrate that engineered amino acid tags introduce to the N- and C-termini of proteins enables their covalent crosslinking.
• Enzymatically crosslinked proteins, notably enzymes in biochemical pathways, can be coupled for efficient metabolic flux.
• Data indicate that varying enzyme stoichiometry in assembled protein complexes can in a directed manner, control metabolic flux.
• Proteins consisting of enzymes, linkers, and recognition domains can be combined for multifunctionality.

## 1 Data

**Fig. 1:**
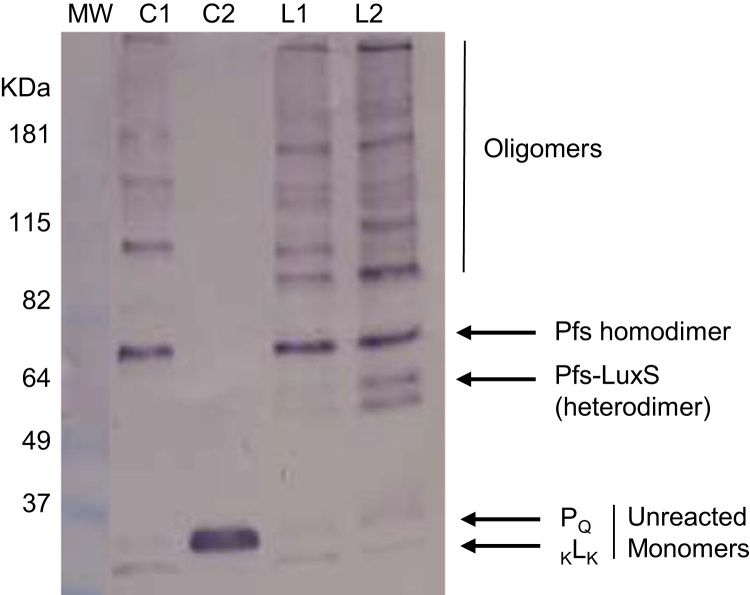
Western blot indicating the differences in crosslinking of L_K_ and _K_L_K_ to P_Q_. Lanes C1 and C2 indicate controls. C1 is P_Q_ assembled on the bead and crosslinked with mTG. Lane C2 is P_Q_ with L_K_ without mTG. Lane L1 is P_Q_ crosslinked to L_K_ using mTG. Lane L2 is P_Q_ crosslinked to _K_L_K_ using mTG. Arrows indicate the monomers, dimers and trimers formed. MW indicates molecular weight. Pfs assembled on the bead is engineered with His tag at N termini. Anti-His antibodies are used in western blots to identify Pfs and Pfs linked structures.

**Fig. 2:**
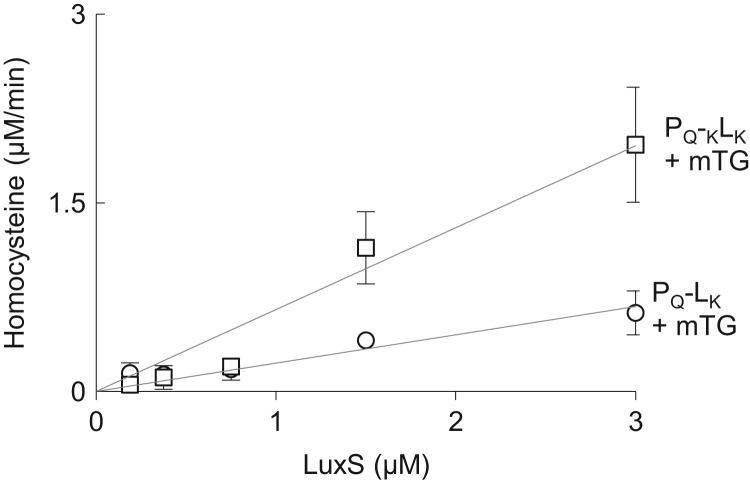
Differences in crosslinking between L_K_ and _K_L_K_ to P_Q_. P_Q_–_K_L_K_ and P_Q_–L_K_ complexes are assembled on beads and incubated with 1 mM SAH for 1 h at 37 °C. Homocysteine generated is measured by Ellman’s Assay after 1 h. Error bars indicate standard deviation with *n*=3. Regressed lines included denote linear best fit.

**Fig. 3:**
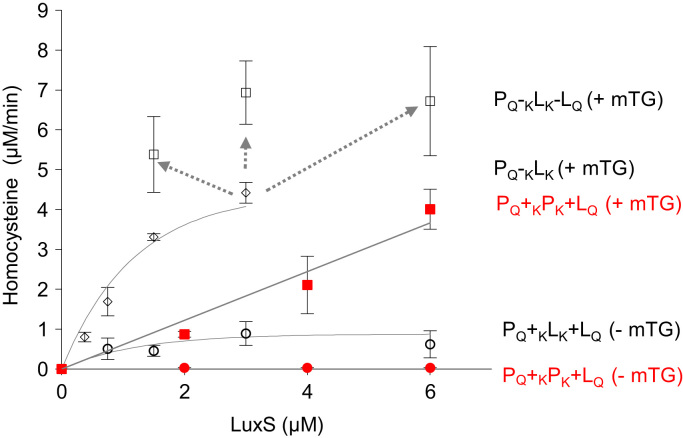
Construction of Pfs–Pfs–LuxS (P_Q_+_K_P_K_+L_Q_) complexes. Red labels indicate HCY yields from Pfs–Pfs–LuxS complexes and data points are juxtaposed onto Fig. 3D [1]. Red squares indicate HCY yields from experimental samples and red circles indicate HCY yields from corresponding mTG- controls. This data supplements Fig. 3E in [1] where Pfs-*Inactive* LuxS–LuxS complex is depicted. Both complexes display similar behavior in HCY kinetics. Three subunit complexes are built onto two subunit complexes constructed with 3 µM. HCY yields are plotted against LuxS concentrations added. Assembled complexes were incubated with 1 mM SAH for 60 min and HCY was measured using Ellman’s assay. Error bars indicate *n*=3.

**Fig. 4:**
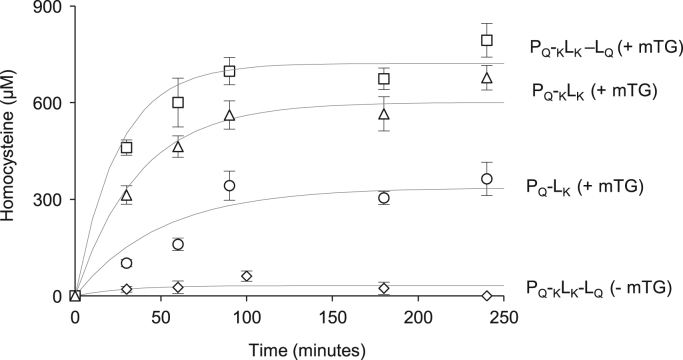
Time course measurements of homocysteine from two subunit (P_Q_–L_K_ and P_Q_–_K_L_K_) and three subunit (P_Q_–_K_L_K_–L_Q_) complexes measured by Ellman’s Assay. Error bars indicate standard deviation with *n*=3. Trend lines denote non-linear regression fit using equation *y*=*y*_max_(1−exp(−*kx*)).

**Fig. 5:**
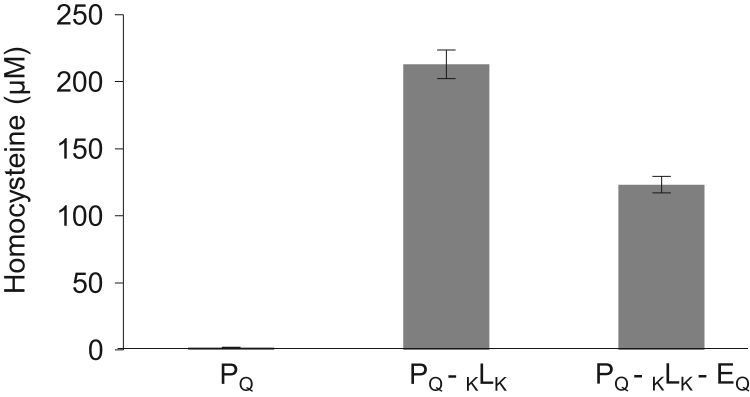
Addition of EGFP leads to decrease in enzyme activity of Pfs–LuxS complex. Assembled protein complexes (Pfs–LuxS and Pfs–LuxS–EGFP) were incubated with 1 mM SAH for 2 h at 37 °C and HCY generated was measured using Ellman’s assay. Error bars indicate standard deviation with *n*=3.

**Fig. 6:**
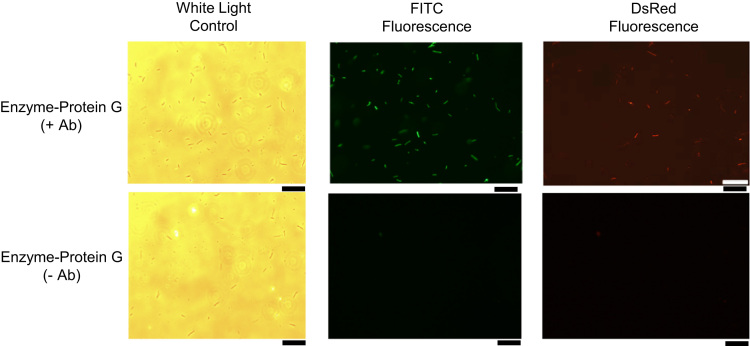
Fluorescence microscopy images of DsRed protein expression in *E. coli* CT104 reporter cells used in Fig. 4E. Top row images: Cells incubated with Enzyme-Protein G complexes coupled with antibodies. Bottom row images: Cells incubated with Enzyme-Protein G complexes without antibodies. Antibodies are labeled with FITC. Scale bar: 50 µm.

Western blot data (Fig. 1) indicated the size and distribution of protein complexes constructed using the modular construction approach. Metabolic flux data indicated the role of engineered tags (Fig. 2), stoichiometries (Fig. 3, Fig. 4), and non-enzyme components (Fig. 5) on activities of complexes constructed. Fluorescence microscopy images (Fig. 6) indicated the response of bacteria to AI-2 generated by Pfs and LuxS enzymes in Pfs–LuxS–Protein G complexes assembled onto bacterial cells.

## 2. Experimental design, materials and methods

### 2.1 Construction of protein complexes on beads

We engineered the first protein component (Pfs) to contain an His tag at the N terminus and a Gln tag at the C terminus and immobilized it to His-affinity beads. We engineered the second component (LuxS) with Lys tags at either the C terminus or both the N and C termini. We crosslinked the second component to the first by addition of enzyme microbial transglutaminase (mTG) and incubated them for 60 min at RT. Post-incubation, we washed the beads 3x with PBS to remove unreacted components and mTG. In the case of the three protein complex construction, we repeated the crosslinking process using a third protein component.

### 2.2 Western Blotting to analyze crosslinking efficiencies

We eluted the crosslinked complexes from beads by the addition of 200 mM imidazole and subjected the complexes to western blotting using standard protocols.

### 2.3 Metabolic flux measurements

We incubated the assembled Pfs–LuxS enzyme components with Pfs substrate SAH at 37 °C for either 60 mins (in Fig. 2, Fig. 3, Fig. 5) or over varying periods of time up to 240 min (Fig. 4). Post-incubation, the amount of homocysteine, HCY, generated was measured using the Ellman׳s DTNB (sulfydryl׳s) assay.
