# Supplementary material for: Data on biochemical fluxes generated from biofabricated enzyme complexes assembled through engineered tags and microbial transglutaminase
Source: Data Brief. 2016 Jul 9;8:1031–5. doi: 10.1016/j.dib.2016.07.005 (PMC4969242; doi:10.1016/j.dib.2016.07.005)
Supplement: Supplementary file 1 — Supplementary material [file mmc1.docx]

*Data article*

**Title:** Data on biochemical fluxes generated from biofabricated enzyme complexes assembled through engineered tags and microbial transglutaminase.

**Authors:** Narendranath Bhokisham^a,b^, Haig Pakhchanian^c^, David Quan^b,c^, Tanya Tschirhart^b,c^, Chen-Yu Tsao^b,c^, Gregory F. Payne^b,c^, William E. Bentley ^a,b,c^.

**Affiliations:**

a: Biological Sciences Graduate Program - College of Computer, Mathematical and Natural Sciences, 4066 Campus Drive, University of Maryland, College Park, MD 20742.

b: Institute of Bioscience and Biotechnology Research, College Park, 5115 Plant Science and Landscape Architecture Building, University of Maryland, College Park, MD 20742.

c: Fischell Department of Bioengineering, Room 3122, Jeong H. Kim Engineering Building (Bldg. #225), University of Maryland, College Park, MD 20742.

**Contact email:** [bentley@umd.edu](mailto:bentley@umd.edu)

**Conflict of Interest**

We have no conflicts, implied or otherwise, with publication of this work.
